# Dental Hygiene for Children*Extract from President’s address in Illinois State Society, published in *Journal N. D. A.*

**Published:** 1920-10

**Authors:** G. Walter Dittmer


### DENTAL HYGIENE FOR CHILDREN*

BY G. WALTER DITTMER, D.D.S.

Much harm often is done in the mouths of children before they attain school age, and it would be most desirable to administer to these children when very young—yes, even before they are born, by caring for the mouths of their mothers. How can a neglected mother whose system is infected from unhygienic oral conditions, who can not properly masticate her food, who is anemic and diseased, be expected to produce strong and healthy children?

While, no doubt, the oral hygiene propaganda which is spreading the gospel of clean mouths by means of the various agencies before mentioned, will promote to a very great extent the welfare of the people—still the public school is undoubtedly the most direct and most effective avenue through which the desired results will eventually be obtained.

It is a laudable fact that for some years past some attention has been paid to the physical welfare of the children in the public schools, especially in the larger cities. The health departments of such cities have provided physicians and nurses, and in some instances they have also provided dentists to examine the mouths and render dental service.

---

*Extract from President's address in Illinois State Society, published in *Journal N. D. A.*

Some of our larger cities have conducted free dental infirmaries, but the children in the smaller cities and towns, and those residing in the rural districts are not favored with such service.

Our states could render no greater service to its future men and women than to provide properly regulated compulsory dental examinations for every child of school age, and for the indigent poor provide in every community a means by which such children will receive sufficient attention to make them as nearly as possible healthy and useful citizens.

The state of Kansas, which is a leader in progressive movements, has recently placed in its statute books a law making the examination of the teeth of all its school children compulsory.

Dr. Florence B. Sherbon, director of Child Hygiene of the state of Kansas, says:

"The law requiring free dental inspection in all the public schools of Kansas makes possible four important results.

"1. Immediate benefit to the health of the children treated.

"2. Effective education of the children in practical dental hygiene.

"3. The securing of statistics which will reveal present conditions.

"4. The compilation of data for a basis for future methods of procedure."

Concluding the article recently published in the *Bulletin* of the Kansas State Board of Health, she further says: "When the results of this combined dental and physical inspection of the school children of Kansas are summed up, it will make one of the most valuable pieces of health research undertaken in any state and will furnish unique and important data which will serve as a guide in future health work."

Why should not the great and wealthy state of Illinois enact a similar law? There can be no question but that every state in the Union should. It would be the most paying investment this nation ever made. Your President, therefore, recommends that the Committee on Legislation of this Society prepare and present to the Illinois legislature at its next session, a bill providing for free dental examinations for every child in the public schools in the state. Certainly the value of such a bill is evident, and we believe it would pass the legislature unanimously. (The support the legislature has accorded our profession in matters of dental legislation in recent years is highly commendable—the reason being that we have been unselfish in our demands and asked only for measures which were fair and just.) Certainly our profession could not be indicted for fostering selfish motives when it is so evident that mouth examinations, mouth hygiene teaching, and proper dental care given to children will prevent the train of so serious, dangerous and costly consequences.

*What must we, as a profession do to prepare ourselves properly to meet the demands which will be made of us?* As I view it, there are a number of things which must be done conjointly with the enactment of the desired law above mentioned. The *first* of these is to *standardize oral hygiene teaching in our dental colleges.* That should really be done throughout the entire nation. Some competent authority should be delegated to write and have properly illustrated a textbook on the "Care of the Teeth and Mouth." This should be done under the direction of the Oral Hygiene Committee of the National Dental Association. *Second,* this textbook should be adopted by the American Institute of Dental Teachers and used in every dental college in America. *Third,* a bill providing for the educating and licensing of dental hygienists who shall be so educated that they will be well qualified to properly examine and clean teeth, and to teach individuals how to practice oral hygiene. Such dental hygienists should be placed in the various public and private institutions that are approved by the Department of Registration and Education. Their activities in these institutions should, of course, be supervised by licensed dentists, or a commission which includes a licensed dentist so that proper service will be rendered.

A number of states now have laws, licensing dental hygienists—viz.: New York, Massachusetts, Connecticut, Oklahoma, Michigan, Minnesota and Colorado. And there may be others.

Another potent agency, it seems to me, which our Public Service Commission should interest in this cause, is the office of the Superintendent of Public Instruction. Through that office let there be instituted a campaign to instruct the public school teachers. This could be done at teachers' institutes and teachers' meetings by supplying literature and lecturers on mouth hygiene. Every teacher in the state should be instructed in the details of mouth hygiene and compelled to give the subject attention in the classroom. This would have a wholesome influence on the children. The placing of a lesson in each public school reader—very simple and plain in the first reader, and more advanced in each succeeding year—would aid in keeping the subject before the child and with supplementary instruction given by the teacher, and occasionally by a dental hygienist or dentist, the child would be so impressed that he would intelligently practice mouth hygiene.

The younger the child who is taught how properly to clean his teeth, who unfailingly and systematically cleanses his teeth, the less harm will result to the teeth, to the surrounding tissues, and to the child's general health.

A logical campaign of action for caring for children under school age as well as for those of school age was presented in a report prepared by the Public Service Commission of the Chicago Dental Society, and was submitted to the Committee on Health of the Chicago Board of Education. The report bears date of April 21, 1919. The Commission consisted of Drs. Thomas L. Gilmer, Truman W. Brophy, C. N. Johnson, Arthur D. Black, and James P. Smith, chairman. The report deals in a thorongh and exhaustive manner with the needs of dental care for the children of Chicago and the surrounding country, pointing out a plan of procedure and giving consideration to the probable financial requirements, and is the method that was recommended for the care of these children that I desire to submit:

FIRST

For children under six years of age, secure through the co-operation of the Child Welfare and other social service agencies of Cook County, and the Public Service Commission of the Chicago Dental Society, every organization concerned with children under six years of age, to be utilized in a campaign of education and instruction in mouth hygiene.

1. By the establishment of pre-school health centers, in every-day nursery, community center, etc. Dental hygienists should examine and clean the teeth of the children, teach mouth hygiene, give toothbrush drills, etc. Reparative dental service should also be arranged for.

2. By the liberal use of educational literature, to inform parents of the importance of care of the temporary teeth, and to urge that mouths be examined by practicing dentists or dentists at day nurseries.

3. By distributing similar literature to children in the public schools to be taken home so that mothers of younger children will be reached. Money so expended will result in much economy in the care of the mouths of these children after they enter public schools.

4. By public lectures in community centers and public schools, these to be part of a general scheme of health lectures.

SECOND

For children in the public and parochial schools.

(a) *Prevention.*

1. Thorough examination of the mouths of all the children at least twice a year.

2. Cleaning the teeth of all the children by dental hygienists at least twice a year.

3. Require each child to have a toothbrush. Supply brushes free to all who can not buy them.

4. Toothbrush drills and short talks by dental hygienists.

5. Distribution of literature for education of children and their parents relative to the importance of proper care of the teeth and the consequences of lack of care.

6. Public lectures to both children and parents in the school buildings, these to be a part of the general scheme of health lectures.

(b) *Dental Care.*

1. Memoranda to parents of services needed, urging treatment.

2. Classification of dispensary and non-dispensary cases.

3. Free dental service for those who can not afford to pay.

THIRD

*For Rural Schools in Cook County.*

Same provisions as for Chicago schools; the dental service being given by an automobile dispensary. Regular dental clinics should be established in the health centers conducted by the Public Health Division of the County Bureau of Social Service. Dentists in these districts should, for a time at least, give gratuitous service in co-operation with the service contributed by physicians.

FOURTH

*Statistics.*

1. A scheme of records should be worked out by which the dental examinations and records would become a part of the medical examinations and records of the child and yet keep a separate (duplicate) record for use by the Dental Service.

2. The scheme should provide for a record of each child in mouth hygiene (to be entered on the regular scholastic report card if practicable); also of the mouth condition each year, and the treatment, all in such form as could be tabulated with reference to the child's general, medical, scholastic and attendance record.

This, it would seem, is a well-digested plan, and it is earnestly hoped it will be adopted. It also suggests a working basis for the smaller cities and towns and rural districts whose needs are more urgent than are those of the larger cities.

Results which have been obtained in numerous experiments made in the public schools throughout several of the states where the improved health conditions and decided advance in scholarship made by groups of children that had received proper dental care compared with similar groups of children that had not been so cared for, convincingly demonstrated the fundamental importance of oral hygiene as a decided economic, as well as a public health measure.

I therefore urge that the organized dental profession of this state be a unit in supporting legislation which will create properly educated and licensed dental hygienists that may be employed by the authorities of schools, hospitals and other institutions, whether public or private, which are approved by the Department of Registration and Education. Also create and maintain free dental inspection of the children in the public schools in this state along lines similar to House Bill No. 323 enacted recently by the state of Kansas.

